# Prevalence of human papillomavirus (HPV) in the semen of patients submitted to assisted reproductive technology treatment in a private clinic in Brazil

**DOI:** 10.5935/1518-0557.20190009

**Published:** 2019

**Authors:** Renata de Lima Bossi, Jéssica Bruna Fernandes Valadares, Helen Lima Del Puerto, Maria Gabrielle Lima Rocha, Letícia Conceição Braga, Marcos Aurelio Coelho Sampaio, Patrícia Pinho França, Débora Moreira Alvarenga, Selmo Geber

**Affiliations:** 1 Centro de Medicina Reprodutiva- Belo Horizonte/MG- Brazil; 2 Departamento de Patologia Geral do Instituto de Ciências Biológicas da Universidade Federal de Minas Gerais- Brazil; 3 Departamento de Análises Clinicas e Toxicológicas da Faculdade de Farmácia da Universidade Federal de Minas Gerais - Brazil; 4 FUNED - Fundação Ezequiel Dias- Belo Horizonte/MG- Brazil

**Keywords:** HPV, infertility, semen, motility

## Abstract

**Objective:**

The aim of our study was to identify the prevalence of HPV in the semen of men submitted to ART treatment and look into the possible impacts of the virus on sperm parameters.

**Methods:**

Thirty-five patients treated for infertility from March to August 2016 were invited to join the study. Samples with a minimum concentration of 40x10^6^ spermatozoa per milliliter were included in the study. After the evaluation of semen parameters, DNA extraction and PCR were performed to verify the presence of HPV by electrophoresis in 8% polyacrylamide gel.

**Results:**

Patient age ranged from 27 to 68 years (mean 39.2 years). Semen analysis showed a mean volume of 2.5mL; mean concentration of 58.9x10^6^; and mean motility of 51.8%. HPV DNA was identified in seven semen samples from 25 patients (28%). Ten samples with DNA concentrations below 10ng/µL were excluded from the study due to poor amplification quality. There was no statistical difference in sperm concentration when HPV-negative and HPV-positive samples were compared (65.9x10^6^ vs. 62.3x10^6^; *p*=0.70). However, sperm motility was significantly higher in HPV-positive semen (65% vs. 46.6%; *p*=0.02).

**Conclusions:**

HPV prevalence was 28% in the semen of patients submitted to ART treatment. HPV-positive samples had statistically increased motility compared to negative samples (65% vs. 46.6%; *p*=0.02).

## INTRODUCTION

Although several human papillomavirus (HPV) types have been identified in semen, the consequences and potential mechanisms linked to alterations in sperm parameters are unknown (Lai *et al*., 1997; Connelly *et al*., 2001; Rintala *et al*., 2004; Foresta *et al*., 2010; Schillaci *et al*., 2013; Yang *et al*., 2013; Golob *et al*., 2014). The impacts of HPV on assisted reproduction technology (ART) treatment and embryo development are under investigation (Brossfield *et al*., 1999; Connelly *et al*., 2001; Lee *et al*., 2002; Foresta *et al*., 2010; Foresta *et al*., 2011a; Garolla *et al*., 2012; Hong *et al*., 2013; Foresta *et al*., 2015; Lyu *et al*., 2017). According to the WHO guidelines (2016), HPV is one of the most important causal factors associated with cancer in women worldwide, with approximately 150 genotypes described (Foresta *et al*., 2015). Considering male infection, many studies have demonstrated associations between HPV and penile, anal, rectal, and oropharyngeal cancer. HPV may also be present in the semen of asymptomatic men (Lai *et al*., 1997; Connelly *et al*., 2001; Rintala *et al*., 2004; Foresta *et al*., 2010; Foresta *et al*., 2011b; Yang *et al*., 2013; Schillaci *et al*., 2013; Garolla *et al*., 2013; Foresta *et al*., 2015; Golob *et al*., 2014; Garolla *et al*., 2016).

Semen analysis is the most important element in the assessment of male factor infertility. Complementary tests for infectious diseases such as Syphilis, HIV 1 and 2, Hepatitis B and C, HTLV I and II, *Chlamydia trachomatis*, *Ureaplasma urealyticum, Mycoplasma hominis*, *Neisseria gonorrhoeae*, aerobic bacteria, and Zika virus are performed routinely before ART treatment in Brazil. Nevertheless, more specific tests such as HPV are not performed.

The prevalence of HPV in semen has been estimated and varies according to the subjects and the country of study. Chan *et al*. (1994) found infection by HPV in 35.7% of the analyzed sperm samples. Foresta *et al*. (2010) found the lowest prevalence among studies involving infertile patients, with 10.2%. Perino *et al*. (2011) looked into a cohort of 199 sperm samples from infertile couples and found that 9.5% were infected by HPV. Schillaci *et al*. (2013) also studied infertile couples and reported a prevalence of 7.8% of infection by HPV in a set of 308 samples analyzed. Yang *et al*. (2013) described the presence of HPV in 107 of 615 (17.4%) infertile patients. Laprise *et al*. (2014) described a prevalence of 16% of HPV in the semen of men with unexplained infertility. Garolla *et al*. (2016) described the presence of HPV in 23.9% (54 of 226 patients) of infertile patients submitted to ART treatment.

The associations between HPV and semen analysis results are still controversial. Many studies have shown evidences of a negative correlation between HPV and semen analysis (Lai *et al*., 1997; Lee *et al*., 2002; Foresta *et al*., 2010; Garolla *et al*., 2013; Garolla *et al*., 2016). However, several studies did not observe differences on sperm parameters between HPV-positive and HPV-negative samples (Connelly *et al*., 2001; Rintala *et al.*, 2004; Schillaci *et al*., 2013; Golob *et al*., 2014). Some studies have suggested that the presence of HPV might induce sperm DNA fragmentation (Connelly *et al*., 2001; Lee *et al*., 2002; Hong *et al*., 2013), disrupt the ability of spermatozoa to bind to and penetrate the oocyte (Foresta *et al*., 2011a), impair embryo development and blastocyst implantation (Gomez *et al*., 2008; Foresta *et al*., 2011b; Gizzo *et al*., 2013), and increase miscarriage rates (Perino *et al*., 2011).

Among the possibilities to treat infected sperm samples, Garolla *et al*. (2012) proposed various sperm washing techniques and showed that a modified swim-up plus heparinase III successfully removed HPV DNA form sperm surface. So far, there is no consensus as to whether HPV truly impairs sperm function. Moreover, the actual prevalence of HPV on semen is still unknown. Therefore, our study aimed to identify the prevalence of HPV in the semen of men submitted to ART treatment and to identify the possible impacts of the virus on sperm parameters.

## MATERIALS AND METHODS

We performed a prospective study analyzing semen samples of men submitted to ART treatment at the ORIGEN Center for Reproductive Medicine from March to August 2016. A total of 35 patients agreed and gave informed consent to join the study.

Samples were collected by masturbation after an ejaculatory abstinence period of two to five days. After 60-minute liquefaction, the samples were analyzed for macroscopic (volume, pH, viscosity) and microscopic (concentration, motility, and morphology) parameters. Concentration and motility analysis were performed using phase contrast microscopy (Nikon - Diaphoto - Japan), a differential cell counter, and a Makler chamber (Origio, Dinamarca).

After ICSI, DNA extraction from the semen samples was performed using a commercially available Wizard^®^ Genomic DNA Purification Kit (Promega) according to the manufacturer's protocol. Flushing was performed initially to remove cells that were not targeted by the present study. Approximately 40x10^6^ spermatozoa from each patient were used.

The total extracted DNA was quantified in an automatic NanoVue Plus Spectrophotometer (GE) and the total DNA concentration was estimated considering that an optical density unit (OD) corresponds to a concentration of 0.050µg/µL of double stranded DNA (dsDNA) (Moore *et al*., 1997).

The detection of HPV genome in semen samples was performed with polymerase chain reaction (PCR) using primers GP5+ (5'-TTTGTTACTGTGGTAGATACTAC-3') and GP6+ (5'-GAAAAATAAACTAACTGTAAATCATATTC-3'), which amplify approximately 150 bp fragments of the L1 viral gene and allow the amplification of all HPV subtypes. PCR reactions were performed using the GoTaq^®^ Green kit (Promega) according to the manufacturer's protocol without modifications, following the concentrations and conditions for PCR.

PCR products were observed on 8% polyacrylamide gel impregnated with silver nitrate, developed and documented with images. Samples that amplified the fragment of 150 HPV base pairs were considered positive for HPV.

## RESULTS

**Figure 1:**
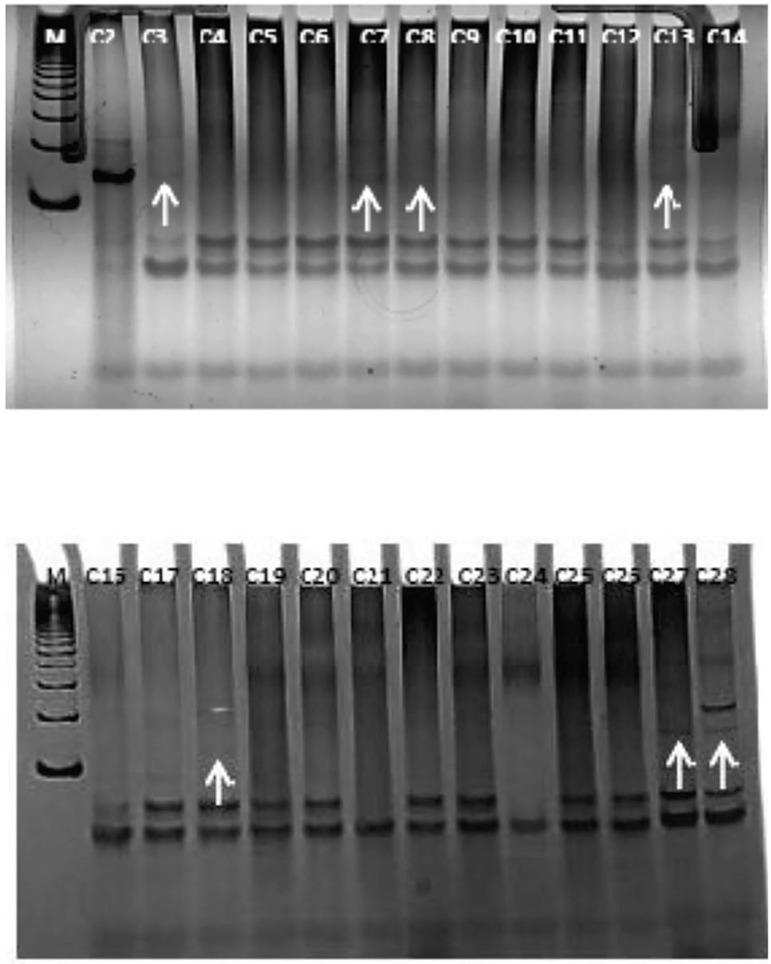
Polyacrylamide gel (8%) stained with silver nitrate. HPV-L1 gene amplification result (~ 150 bp). M = 100 bp molecular weight marker, samples from 1 to 25 = tested semen samples. Arrows marks HPV-positive samples

A total of 35 semen samples were studied. Patient ages ranged from 27 to 68 years (mean=39.2±8.36 years). All patients were evaluated for sexually transmitted diseases and one was positive for HIV. Ten samples with DNA concentration below 10ng/µL of DNA were excluded from the study due to losses in amplification quality. Twenty-five samples with concentration greater than 10ng/µL were submitted to PCR. Seven of the 25 semen samples were positive for HPV (Table 1, Figure 1).

**Table 1:** Semen parameters from 25 patients samples with semen analyzed for HPV prevalence

Patients	Concentration (x10^6^ )	Age	Volume (mL)	Progressive motility (%)
**P06 **	51	39	1.7	69
**P07 **	116	35	2.7	61
**P09 **	16.6	45	0.9	84
**P10**	120	46	0.8	46
**P11 **	31.8	32	2.0	81
**P12 **	153	68	7.2	25
**P13 **	67	38	2.1	59
**P14**	49	43	2.0	51
**P15**	36	36	2.0	26
**P16**	28.3	36	2.0	51
**P17**	37.3	36	4.5	41
**P18**	17	45	1.7	56
**P19**	83.5	44	2.7	62
**P20**	13	34	2.2	11
**P21**	45	33	2.0	68
**P22**	33	27	3.0	64
**P23**	82	32	2.5	67
**P25**	100	28	3.1	68
**P26**	68.5	49	1.4	53
**P27**	89	39	2.0	60
**P29**	64	38	2.0	44
**P30**	64	34	2.0	37
**P31**	41	43	2.0	29
**P32**	31	35	3.7	63
**P34**	35.6	46	5.2	36

Mean sperm concentration was 58.9x10^6^ (16x10^6^ to 153x10^6^). Mean progressive motility (AB) was 51.8% (26% to 81%).

When we compared the sperm concentrations of the HPV-positive and HPV-negative samples, we noticed that both presented similar results, with 62.3% and 65.9%, respectively (*p*=0.70). However, when we compared them for sperm motility, the mean motility of HPV-positive samples was statistically higher than the mean motility of HPV-negative samples (65% vs. 46.6%) (*p*=0.02) (Table 2).

**Table 2:** Statistical analysis of concentration and motility between HPV-positive and HPV-negative semen samples

Semen parameters	All patients (n=25)	HPV+ (n=7)	HPV- (n=18)	*p* Fisher’s test
Concentration (x10^6^)	58.9 (13-153)	62.3	65.9	0.70
Progressive motility (%)	51.8 (26-81)	65	46.6	0.02

The differences in sperm motility and concentration between the HPV-positive and HPV-negative groups were evaluated through Fisher’s exact test on SPSS (version 22.0.0.0).

## DISCUSSION

Our study revealed a high prevalence of HPV (28%) in the semen of infertile couples submitted to ART treatment. Moreover, the HPV-positive samples did not present abnormal parameters according to the WHO (2010) criteria. Therefore, we could not confirm the existence of a correlation between HPV and sperm quality. To our knowledge, this is the first study held in Brazil about HPV prevalence in patients submitted to ART treatment.

The prevalence observed in our study was higher than the prevalence reported by Yang *et al*. (2013), who described the presence of HPV in 107 of 615 (17.4%) infertile patients, and by Laprise *et al*. (2014), in a meta-analysis that reported 16% of infection by HPV in patients submitted to ART treatment. Our results were similar to the numbers described by Garolla *et al*. (2016), in a study that reported the presence of HPV in 23.9% (54 of 226 patients) of infertile patients submitted to ART treatment.

The absence of a difference in sperm concentration between HPV-positive and HPV-negative samples was also described in previous studies (Foresta *et al*., 2010; Schillaci *et al*., 2013; Garolla *et al*., 2016). Our study demonstrated a significant increase in sperm motility in the HPV-positive group. Similar results were described by Connelly *et al*. (2001), where higher levels of motility were observed in sperm exposed in vitro to HPV types 16 (48±0.2%), 18 (47.5±0.1%), 31 (55±0.5%), and 33 (48.5±0.7%) compared to samples not exposed to HPV (37.5±0.3%). Other studies did not find differences between HPV-positive and HPV-negative groups. Rintala *et al*. (2004) analyzed the sperm parameters of 65 sperm donors and found no significant difference in sperm motility. Schillaci *et al*. (2013) showed that non-infected and infected sperm samples had similar progressive motility. Golob *et al*. (2014) studied 340 patient samples and did not find significant differences in motility or sperm concentration.

Differently from our study, Foresta *et al*. (2010) reported a statistical decrease in motility in HPV-positive semen (37.7%±16.8 *versus* 53.7%±18.2%, *p*<0.05), while other sperm parameters were similar. Other studies by Foresta *et al*. (2011a;b; 2015) also described decreased sperm motility in HPV-positive samples. Yang *et al*. (2013) observed that even among controls (fertile patients) HPV-positive samples had significant impairments in sperm motility (32.25%±10 vs. 39.22%±12.15, *p*<0.01) and morphology (8.51±4.21 vs. 13.01±4.50, *p*<0.01). Foresta *et al*. (2015) performed a meta-analysis that included 30 studies and 5325 patients. The authors described a higher prevalence of HPV with decreased semen motility among patients with unexplained infertility, in addition to a strong relationship between asthenozoospermia and HPV regardless of virus subtype. Garolla *et al*. (2016) also reported decreased sperm motility in patients with HPV compared to patients without HPV (25.9±16.2% vs. 34.3±17.7%). The authors suggested this was due to the presence of antibodies on sperm surface, since 40.7% of HPV-positive samples had antisperm antibodies versus 10.5% in non-infected samples.

Lai *et al*. (1997) were the first to describe asthenozoospermia in sperm samples of HPV-positive patients with HPV types 16 and 18. Lee *et al*. (2002) also observed that HPV types 6/11, 16, 18, 31 caused decreases in sperm motility. Some HPV types may have direct impact on sperm motility. Lee *et al*. (2002) showed that type 33 did not affect semen parameters. Therefore, it is important to find the type of HPV present in sperm samples.

In the present study, the causes of infertility in the HPV-positive group were ovarian factor and unexplained infertility. Due to the limited size of our sample, we were unable to find an association between the presence of HPV and unexplained infertility. Previous studies attempted to correlate the presence of HPV and sperm function impairment. Several described increased sperm fragmentation (Connelly *et al*., 2001; Lee *et al*., 2002; Hong *et al.,* 2013). Foresta *et al*. (2011a) reported that sperm infected with HPV were less able to penetrate oocytes since the virus was located on sperm surface. Embryo development may also be affected by HPV (Foresta *et al*., 2011b; Gomez *et al*., 2008). Garolla *et al*. (2012) proposed various sperm washing techniques to treat infected sperm samples and showed that a modified swim-up plus heparinase III successfully removed HPV DNA form sperm surface. Garolla *et al*. (2013) also showed that after 24 months antisperm antibodies disappeared and sperm motility improved.

Further studies are required with greater numbers of sperm samples to identify HPV virus subtypes and quantify the viral load in order to establish whether HPV might affect sperm parameters or impair fertility. Moreover, it is important to elucidate the mechanism by which HPV might affect sperm parameters and the possible implications in IVF outcomes.
